# Induced Regulatory T Cells Superimpose Their Suppressive Capacity with Effector T Cells in Lymph Nodes *via* Antigen-Specific S1p1-Dependent Egress Blockage

**DOI:** 10.3389/fimmu.2017.00663

**Published:** 2017-06-07

**Authors:** Shuang Geng, Yiwei Zhong, Xiaoyu Zhou, Gan Zhao, Xiaoping Xie, Yechun Pei, Hu Liu, Huiyuan Zhang, Yan Shi, Bin Wang

**Affiliations:** ^1^Key Laboratory of Medical Molecular Virology of MOH and MOE, Fudan University Shanghai Medical College, Shanghai, China; ^2^State Key Laboratory for Agro-Biotechnology, China Agricultural University, Beijing, China; ^3^Tsinghua-Peking Center for Life Sciences; Institute for Immunology, School of Medicine, Tsinghua University, Beijing, China; ^4^Department of Microbiology, Immunology and Infectious Diseases, Snyder Institute, University of Calgary, Calgary, AB, Canada

**Keywords:** inducible regulatory T cell, antigen specific suppression, hilar lymph node, airway inflammation, egress, S1p1

## Abstract

Regulatory T cells (Tregs) restrict overexuberant lymphocyte activation. While close proximity between Tregs and their suppression targets is important for optimal inhibition, and literature indicates that draining lymph nodes (LNs) may serve as a prime location for the suppression, signaling details orchestrating this event are not fully characterized. Using a protocol to enable peripheral generation of inducible antigen-specific Tregs (asTregs) to control allergen-induced asthma, we have identified an antigen-specific mechanism that locks asTregs within hilar LNs which in turn suppresses airway inflammation. The suppressive asTregs, upon antigen stimulation in the LN, downregulate sphingosine-1-phosphate receptor 1 egress receptor expression. These asTregs in turn mediate the downregulation of the same receptor on incoming effector T cells. Therefore, asTregs and effector T cells are locked in these draining LNs for prolonged interactions. Disruption of individual steps of this retention sequence abolishes the inflammation controlled by asTregs. Collectively, this study identifies a new requirement of spatial congregation with their suppression targets essential for asTreg functions and suggests therapeutic programs *via* Treg traffic control.

## Introduction

Regulatory T cells (Tregs) play a crucial role in balancing the activation state of the immune system (1, 2). From two natural developmental origins of Tregs, thymic migrants represent a population of self-reactive T cells that escape negative selection (2–5) and become a steady pool of Tregs available in the periphery, designated as tTregs. Peripheral induction of Tregs (pTreg) is more tuned to local antigenic stimulation and cytokine milieu and is therefore more dynamically regulated (6–8). These two populations together form a major immune suppressive network. TCRs cloned from tTregs cannot support an overrepresentation of these cells in transgenic mice, and the population size of these Tregs is limited to a niche percentage set by other TCR specificities (9). This by design leaves a gap in immune suppression to be filled by pTregs risen in response to environmental factors. TCR specificities of these two populations are mostly non-redundant, suggesting the emergence of pTregs is likely driven by the novel antigens not available in the thymus (10). Functional analyses confirm this sharing. For instance, populating a host with tTregs may to a large extent suppress systemic inflammation, however, local autoimmunity can still take place (10). In some cases, pTreg-mediated suppression appears to be central to the negative feedback to local immune activation, such as in oral and mucosal tolerance (11, 12), and particularly in airway inflammation (13). In experimental settings, signaling *via* TCR coupled to anti-inflammatory cytokines, TGF-β and IL-10, typically leads to the generation of canonical pTregs that share Foxp3 and CD25 expression with tTregs, mostly but not exclusively characterized by the lack of Helios and neuropilin 1 (14, 15). These features have been used by some to distinguish tTregs from pTregs (16, 17). A similar population of Tregs induced by vaccination does not express CD25 but are equally suppressive (18).

Although the exact regulatory mechanisms of Tregs are still being debated, a surprisingly large set of proposed models require spatial proximity between Tregs and their suppression targets, such as those mediated by granzyme-induced cytolysis (19, 20) and inhibitory effects of membrane bound TGF-β (21). A recent discovery of Treg inhibition that centers on indirect suppression *via* dendritic cells (DCs), through Indoleamine 2,3-dioxygenase production (22) and CTLA-4-mediated costimulatory molecule depletion (23), is also based on direct contact. We reported recently that strong binding by Tregs triggers DC cytoskeleton polarization, limiting the DCs ability to engage conventional T cells (24, 25). While cell/cell contact is essential, one less illuminated aspect of Treg inhibition is whether or not the suppression is the same regardless of the location of contact. There is some evidence to suggest that Tregs can function in the parenchyma. For instance, Tregs can mediate tissue repair after drug-induced muscle injury (26). The bulk of literature suggests that apart from inflammatory tissue, lymph nodes (LNs) are also central to Treg suppression. *In vivo* and *in vitro* evidence suggests that in LNs, Tregs show prolonged binding to DCs which limits the DCs mobility and reduces antigen presentation to T cells (27–29). Tregs migrate in a ordered sequence from the circulation to LNs for functional suppression (30). While intuitively LN structures are optimal for maximum engagement and exchange between the suppressor and the suppressed, the mechanistic basis for this retention is not known.

In this report, we describe an egress-blocking mechanism that gathers antigen-induced Tregs and antigen-specific effector T cells (Teffs) in local draining LNs. We previously demonstrated that coimmunization with antigen plus its cognate coding DNA construct induced a set of CD4^+^CD25^−^Foxp3^+^CTLA-4^low^PD-1^low^GITR^hi^ Tregs, designated as antigen-specific Treg (asTreg) here. In an asthma model, these cells expressed inhibitory cytokines including IL-10 and TGF-β and inhibited antigen-specific T cell proliferation in the lung (31, 32). We report here that these asTregs mediate their suppressive function mainly in hilar lymph node (hLN) that drains the lung. Antigenic stimulation of these asTregs downregulates their surface sphingosine-1-phosphate receptor 1 (S1p1), which normally mediates LN egress traffic. The retained asTregs in turn trigger S1p1 downregulation on the incoming antigen-specific Teffs. As a consequence, asTregs not only suppress proinflammatory Teffs in this extended cohabitation but also lock those suppressed cells in LNs until their functional withering. Disruption of this collective retention abolishes the suppressive capacity of these asTregs and exacerbates asthma symptoms. This work therefore reveals a spatiotemporal regulation of Tregs essential for their suppressive capacity.

## Results

### OVA Antigen Plus OVA-Coding DNA As a Treatment Can Reduce OVA-Sensitized Asthma through Tregs

**Figure 1:**
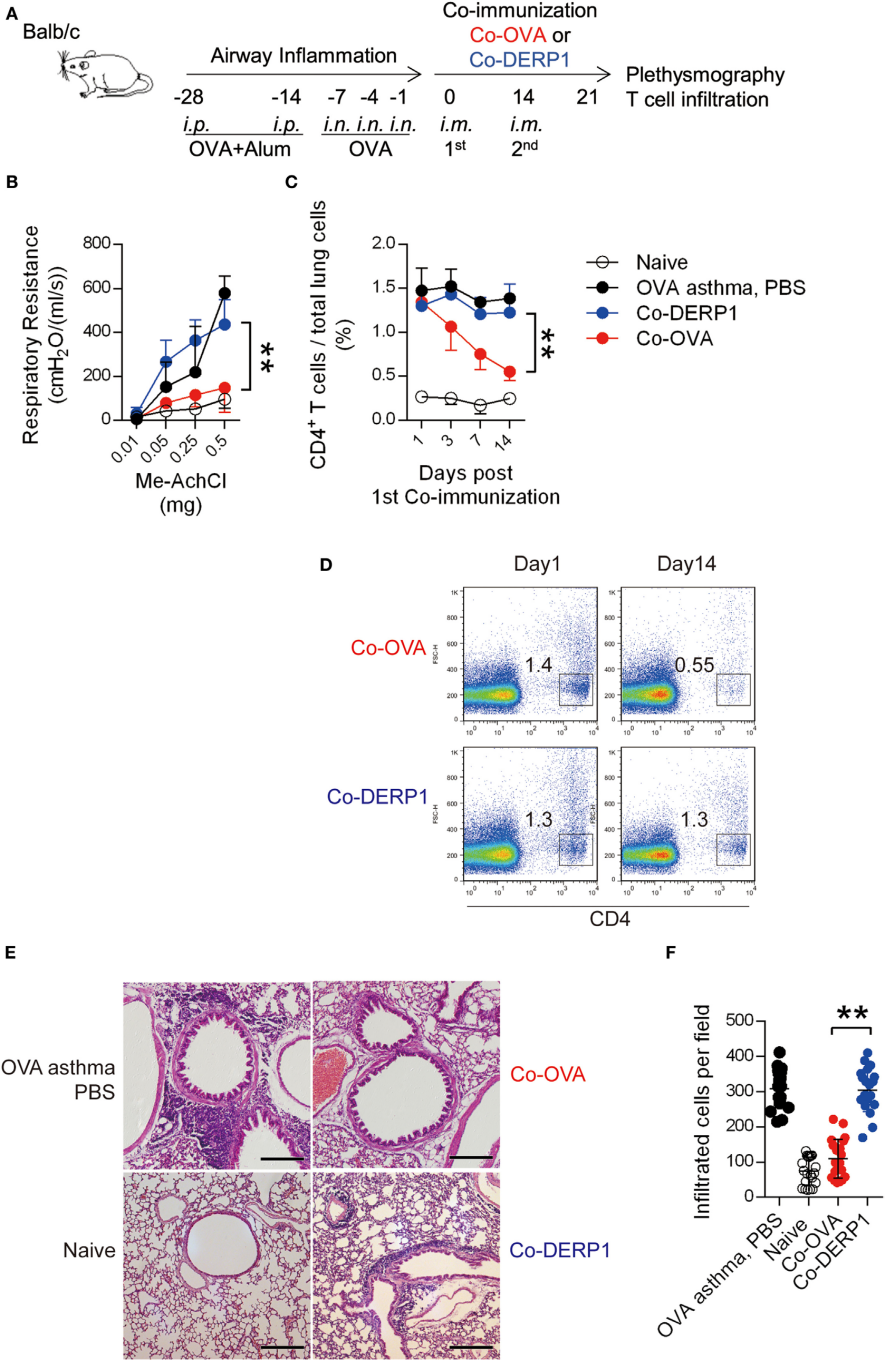
Coimmunization alleviates airway inflammation through regulatory T cells. **(A)** Experimental scheme for coimmunization in the asthma model. Airway inflammation was induced from days −28 to 0 with OVA per marked dates. Mice were then coimmunized on day 0 and 14. Plethysmography and the infiltration were tested on day 21. Both antigen-matched immunization (Co-OVA) and mismatched immunization (Co-DERP1) were performed. **(B)** Plethysmography under methylcholine chloride stimulation. Naïve mice (black circle), PBS (black dot)-, Co-OVA (red dot)-, and Co-DERP1 (blue dot)-treated asthmatic mice are shown. *n* = 4. Same color labeling is used in similar settings there forth. **(C)** Cellular infiltration in lung tissues. Dynamics after coimmunization was analyzed with FACS. *n* = 4. **(D)** FACS data of T cell infiltration in the lung. **(E)** H&E histology of the lung infiltration on day 21. **(F)** Infiltrating cells per H&E slides were analyzed for each group. Every image represents an eye-field under microscope.

We previously developed a vaccine based on coimmunization of OVA peptide (aa323–339 restricted by I-A^d^, recognized by DO11.10 TCR) with a DNA construct carrying the same epitope coding sequence. The immunization induced antigen-specific CD4^+^CD25^−^ asTregs and prevented the host from OVA sensitization-induced asthma (33). To evaluate the therapeutic value of this protocol in established asthma, we induced airway inflammation in BALB/c mice by multi-step sensitization/challenging of intranasal injection of OVA (Figure 1A). The symptomatic mice were then treated with coimmunization of OVA + pVAX1-OVA (Co-OVA) per schedule described in Figure 1A, with antigen-mismatched control treatment of Der p 1 + pVAX1-Der-p1 (*Dermatophagoides pteronyssinus* peptidase 1, Co-DERP1). In the asthmatic mice, respiratory resistance and T cell infiltration in the lung were reduced following the Co-OVA treatment (Figures 1B–D), in comparison with the limited effect of PBS sham or Co-DERP1 control treatment. Histology study showed that inflammatory reactions in the lung were evident in the sham and control treatments, while the Co-OVA ameliorated the inflammatory infiltration (Figures 1E,F).

In Co-OVA-immunized Foxp3-eGFP transgenic mice without asthma induction (scheme in Figure S1A in Supplementary Material), a subset of CD25^−^Foxp3^+^ T cells appeared in CD4^+^CD25^−^ population (9% versus 0.9%) while the size of CD25^+^FoxP3^+^ tTreg population barely changed, suggesting that the Co-OVA protocol preferentially led to CD4^+^ T cell conversion to asTregs, without recruiting or expanding tTreg pool on site (Figures S1B,C in Supplementary Material). As a systematic control for the Co-OVA, Co-DERP1 immunization showed similar induction in the CD4^+^CD25^−^ population rather than the CD25^+^FoxP3^+^ tTreg population (Figure S1C in Supplementary Material). Functionally, the Co-OVA asTregs sorted to homogeneity were able to suppress DO11.10 T cell proliferation in response to OVA stimulation *in vitro* (Figures S1D–F in Supplementary Material). Although any activated T cells or Tregs would compete with the naïve T cells for antigen and available IL-2, as both of them express CD25 and might be antigen-specific, the near absence of any DO11.10 T cell proliferation still suggested an active suppression event.

**Figure 2:**
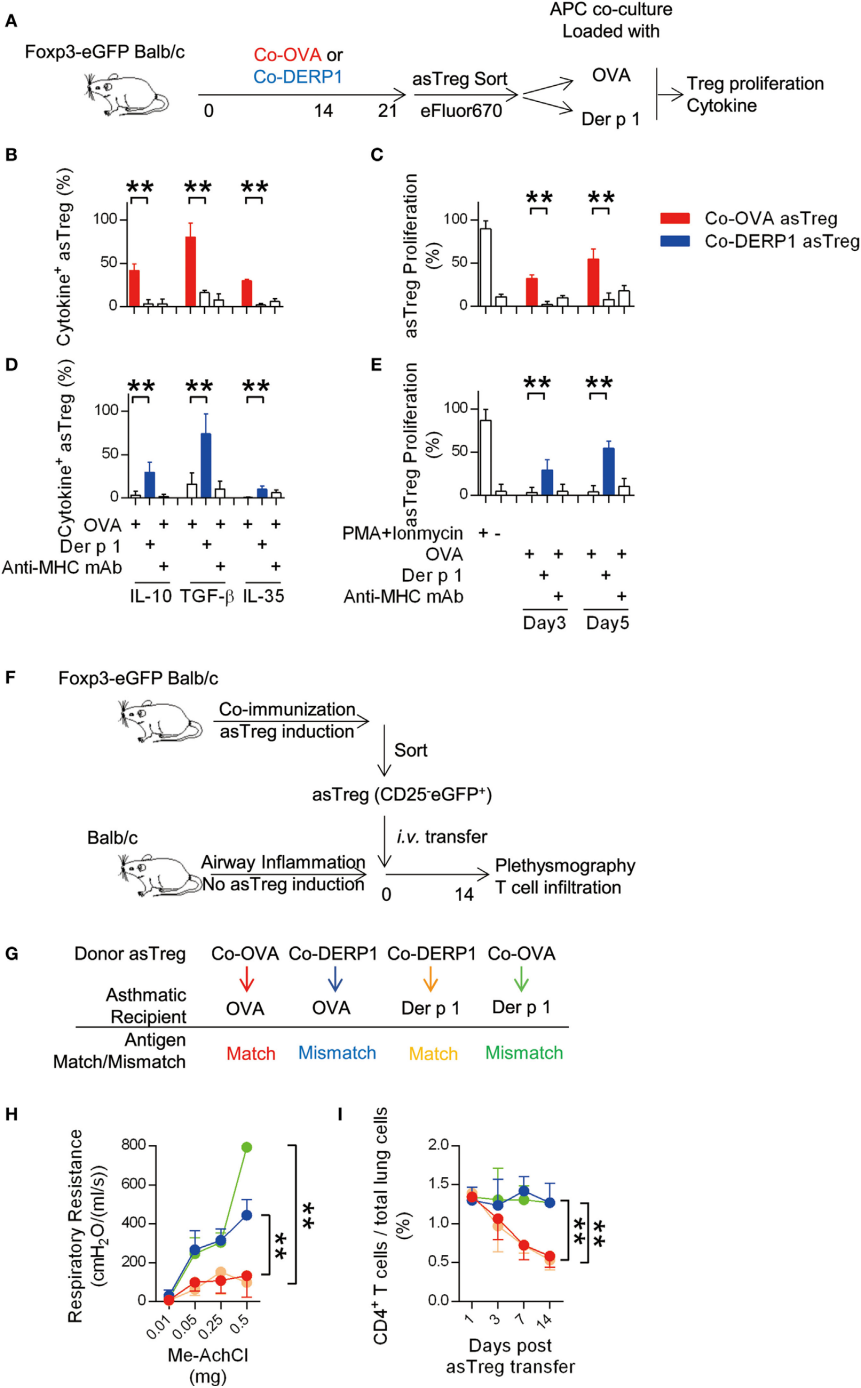
Antigen-specific regulatory T cell (asTreg) transfer ameliorates antigen-matched asthma *in vivo*. **(A–E)** Coimmunization-induced asTregs show specificity *in vitro*. **(A)** Experimental scheme for *in vitro* re-stimulation of sorted asTregs. Foxp3-eGFP Tg mice were immunized on day −21 and −7. On day 0, asTregs were sorted by FACS (Figure S1B in Supplementary Material), labeled and cocultured with antigen-loaded APCs. Both antigen-matched (Co-OVA asTregs with OVA; Co-DERP1 with Der p 1) and mismatched ones (Co-OVA asTregs with Der p 1; Co-DERP1 with OVA) were performed. eFluor670 dilution was analyzed on day 3 and day 5. Cytokine secretion was analyzed 24 h post restimulation by FACS. **(B,C)** Co-OVA asTregs were re-stimulated with OVA or Der p 1. Intracellular suppressive cytokines secretion (b) and Treg proliferation **(C)** were analyzed. Anti-MHC-II mAb was added to block MHC-TCR signal. *n* = 4. **(D,E)** as above, Co-DERP1 asTregs were re-stimulated with OVA or Der p 1. *n* = 4. Data shown represent 3 independent experiments. **(F)** Experimental scheme for asthma treatment by asTreg transfer. **(G)** Antigen-matched and mismatched pairs of asTreg-transfer were performed. **(H)** Respiratory resistance of OVA asthmatic recipient mice (red and blue) or Der p 1 asthmatic recipients (orange and green) were analyzed on day 14 with plethysmography. *n* = 4. **(I)** Dynamics of CD4^+^ T cell presence in lung tissues by FACS. Data shown represent three independent experiments.

To study whether those asTregs responded to the antigen specifically, they were stimulated with OVA or Der p1-loaded APCs *in vitro* (Figure 2A). Co-OVA asTregs upon OVA but not control Der p1 stimulation secreted IL-10, TGF-β, and IL-35 (Figure 2B; Figure S1G in Supplementary Material) and showed robust proliferation (Figure 2C; Figure S1H in Supplementary Material). Those functions were entirely dependent on MHC class II (Figures 2B,C). In a mirror image, Co-DERP1 asTregs responded to Der p1 rather than OVA with a similar spectrum of activations (Figures 2D,E). This proliferation upon cognate antigen stimulation appeared to be substantial in comparison with a more strained antigen-driven division of tTregs reported in some articles (34–37). Our results therefore suggest that asTregs may rise in the presence of overt, specific antigens.

To rule out additional in-host changes that may act as confounding factors in parallel to asTreg induction, we produced Co-OVA or Co-DERP1 asTregs *via* the coimmunization protocol, isolated and transferred them into OVA or Der p 1-sensitized asthmatic mice, thus leaving antigen specificity matching/mismatching between effector T cells and asTregs the only variable in the system (two-step schemes are in Figures 2F,G). OVA asthmatic mice receiving Co-OVA asTreg transfer rather than Co-DERP1 asTreg showed amelioration both in plethysmography readouts and in T cell infiltration. Similar reductions in Der p1 asthmatic mice were only evident with Co-DERP1 asTregs (Figures 2H,I). These data collectively demonstrate that asTregs induced by coimmunization inhibit T cell responses and inflammation in asthma in an antigen-specific manner.

### Accumulation of asTregs in Lung hLN

**Figure 3:**
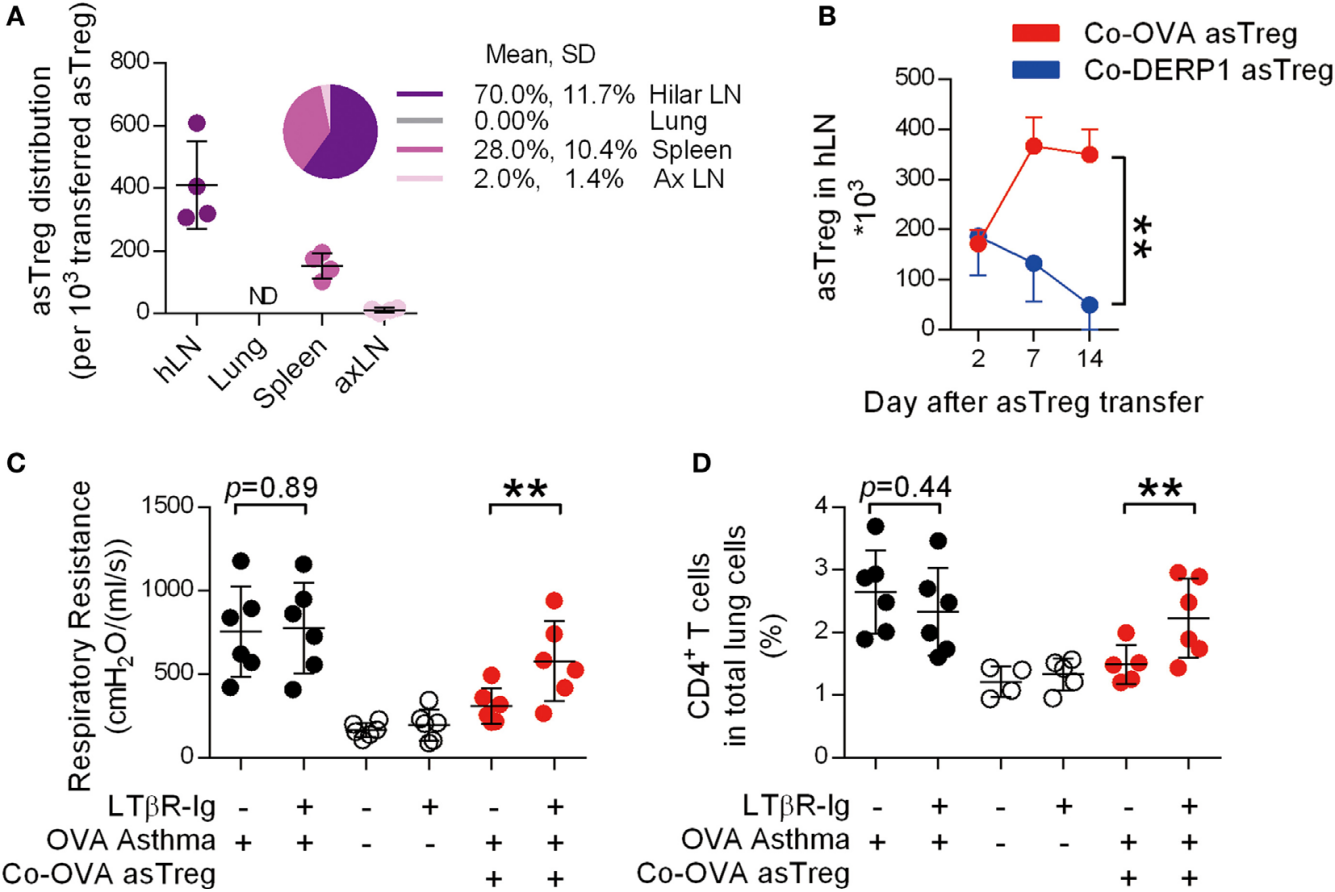
Antigen-specific regulatory T cells (asTregs) accumulate in lung hilar lymph node (LN). **(A)** With the same model as Figure 2F, OVA-induced asthmatic recipient mice were analyzed for eGFP^+^ asTreg in lymphoid organs by FACS, 2 days after Co-OVA asTreg transfer. Shown are eGFP^+^ asTreg distributions in hLN, lung, spleen, and axillary lymph node (axLN). Each spot represents one mouse, *n* = 4. ND, not detectable. **(B)** hLN asTreg kinetics. On days 2, 7, and 14, eGFP^+^ asTregs were analyzed in hLN of Co-OVA asTreg (red line) or Co-DERP1-treated recipients (blue line) by FACS. *n* = 4. Respiratory resistance **(C)** and inflammatory infiltration **(D)** with or without hLN. Asthmatic (black dot), naïve (black circle), and Co-OVA treated (red dot) mice were analyzed. *n* = 6. Data shown represent three independent experiments.

Although there is agreement that Treg-mediated suppression requires close proximity, either to Teff (38–42) or DCs (43, 44), it is less clear where the primary location of such a suppressive function is carried out. We transferred GFP^+^Co-OVA asTregs into recipient OVA-sensitized asthmatic mice and examined the lung and its associated lymphoid organs for GFP^+^ cells 2 days after their infusion. Unexpectedly, measured by total cell numbers recoverable, most of the asTregs were located in the lung-associated hLN, limited numbers of asTregs were found in the auxiliary LN and they were essentially undetectable in the lung (Figure 3A). Visual inspection also revealed an expanded size of the hLN (Figure S2A in Supplementary Material). The exceptionally high recovery of these cells in hLN might be influenced by the prior imprint priming in the donor and their expansion upon antigen exposure in the recipients. We did not pursue these possibilities further. Histology of hLN sections showed that the asTregs distributed in both B cell and non-B cell zones (Figure S2B in Supplementary Material). To follow their dynamic changes in hLN, numbers of GFP^+^ asTregs were continuously monitored over a period of 2 weeks. In our system, both specific and non-specific asTregs trafficked into hLN on day 2. However in hLN, Co-OVA asTregs increased by days 7 and 14 (Figure 3B), whereas Co-DERP1 asTregs faded gradually, suggesting an antigen-driven retention of OVA-induced asTregs.

Draining LNs are the primary location of antigen-specific T cell priming (45, 46). Maternal blocking of lymphotoxin ablates secondary lymphoid organs in the offspring (47). To demonstrate the role of hLN for asTregs to exert their suppression, LN-null mice were generated by maternal injection of LTβR-Ig during gestation. After asthma induction in these LN-null mice, asTregs were adoptively transferred. hLN in mice born following the maternal injection of LTβR-Ig was not visible (Figure S2C in Supplementary Material). Yet, the absence of hLN did not affect the asthma establishment (Figures 3C,D). However, in these mice, infusion of Co-OVA asTregs was no longer able to suppress asthma, measured by both airway restriction and CD4^+^ T cell infiltration into the lung (Figures 3C,D). These results suggest that in our model, the anatomic structure of LNs in general, particularly hLN, is essential for the suppression of T cells; the mere provision of the full complement of asTregs is not sufficient.

### Antigen Triggered Reduction of S1P1 Mediates the Chemotactic Retention of asTregs in hLN

**Figure 4:**
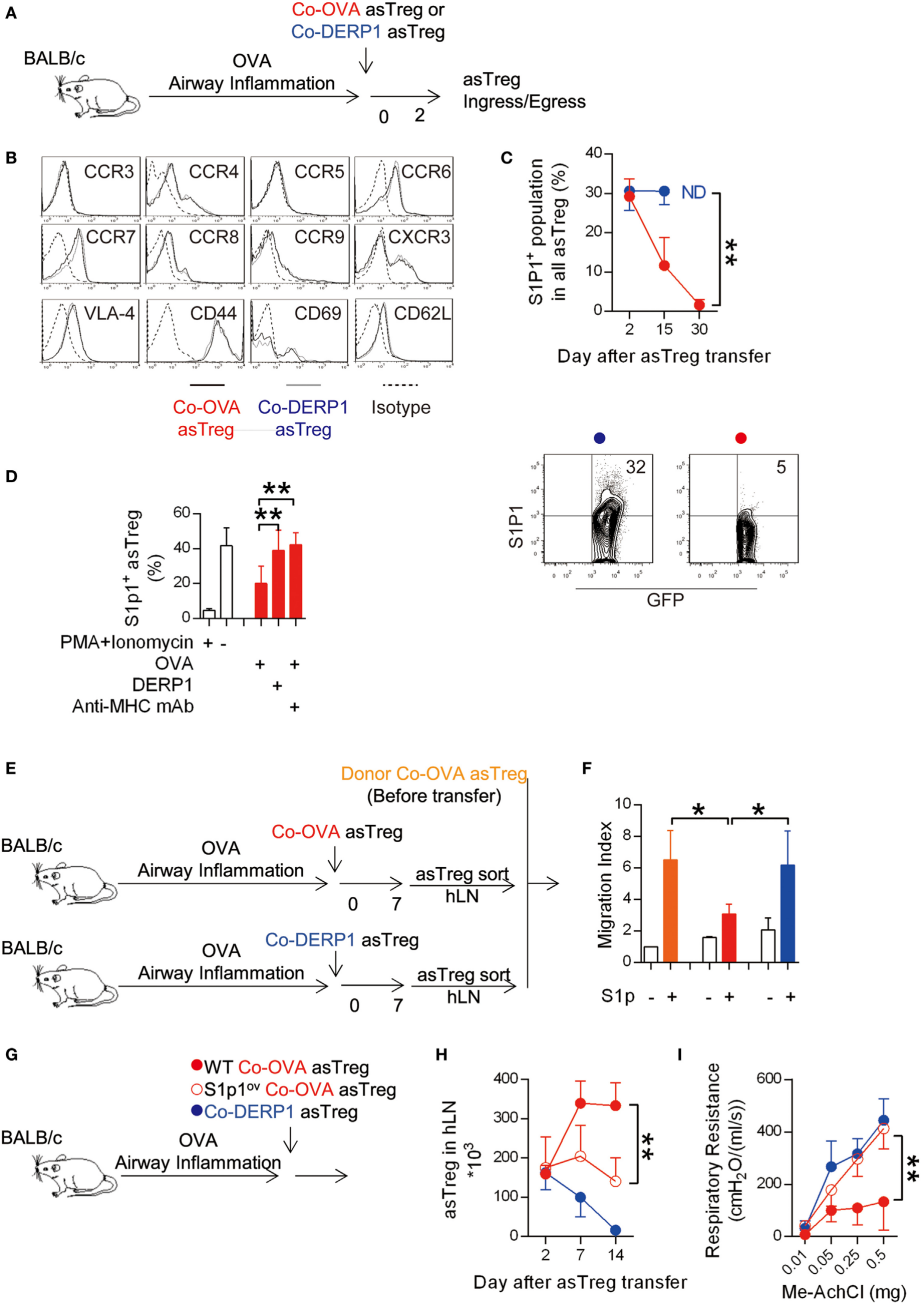
Antigen-triggered S1p1 reduction retains antigen-specific regulatory T cells (asTregs) in hilar lymph node (hLN). **(A)** Similar to Figure 2F. Both antigen-matched (Co-OVA, red) and mismatched (Co-DERP1, blue) treatments by asTregs were performed on OVA asthmatic recipient mice. **(B)** Ingress chemokine receptors and adhesion molecules on hLN asTregs were analyzed 2 days after the transfer. **(C)** Egress chemokine receptor S1p1 on hLN asTregs posttransfer. ND, at day 30, Co-DERP1 asTregs could not be detected in hLN. FACS data showed S1p1 expression on asTregs at day 15. *n* = 4. **(D)** Sorted Co-OVA asTregs were cocultured with antigen-loaded APCs. PMA + Ionomycin were added as positive control. Anti-MHC class II mAb was added to block antigen stimulation to TCR. *n* = 3. **(E,F)** *In vitro* transwell experiment for asTregs’ chemotactic migration toward S1p ligand gradient. Donor asTregs freshly sorted as control (orange). Co-OVA (red) or Co-DERP1 asTregs (blue) recovered from hLN 7 days after the transfer were analyzed. *n* = 3. **(G)** Experimental scheme for S1p1-overexpressing asTreg treatment. **(H)** S1p1 overexpression on asTreg (red circle) showed impaired retention in hLN comparing with the wild type (red dot). *n* = 3. **(I)** Respiratory resistance of OVA asthmatic recipient mice on day 14. Recipient treated with S1p1-overexpressing Co-OVA asTreg (red circle) failed to ameliorate asthma comparing with wild type asTregs (red dot). *n* = 3. Data shown represent three independent experiments.

Lymphocyte ingress and egress with respect to LNs are regulated by chemotactic factors (48–50). The same regulation is also exerted on Tregs (51). To identify any surface molecules that might be responsible for the retention of asTreg, we compared the expression profile of chemokine receptors and adhesion molecules on the antigen-matched asTregs recovered from hLN 2 days after their transfer with those of the mismatched control (Figure 4A). Ingress receptors on Co-OVA asTregs, including CCR4, CCR6, CCR7, CCR8, CCR9, and CXCR3 were found to be expressed at levels similar to those on Co-DERP1 asTregs; nor was any difference detected for adhesion molecules VLA-4, CD44, CD69, and CD62L (Figure 4B). Interestingly, both populations showed no detectable CCR3 and CCR5. However, the expression dynamics of egress chemokine receptor S1p1 on the Co-OVA asTregs showed a precipitous drop after the transfer, in a sharp comparison with the steady expression on Co-DERP1 asTregs (Figure 4C). Antigen-triggered S1p1 regulation helps T cells to localize in the peripheral lymphoid organs (52), however whether it holds true for asTregs *in vivo* is not known. To confirm this central hypothesis, donor Co-OVA asTregs were cocultured with antigen-loaded APCs *in vitro*. As depicted in Figure 4D, expression of S1p1 on the asTregs was significantly reduced only in the culture with OVA-loaded APCs. This reduction was blocked by the addition of anti-MHC-II mAb, suggesting the class II antigen presentation was essential to induce the S1p1-dependent retention of those asTregs.

Transwell experiments *in vitro* with donor Co-OVA asTregs before transfer, and those hLN Co-OVA and Co-DERP1 asTregs recovered day 7 after their transfer into OVA-sensitized mice (without endogenous induction of asTregs by DNA/antigen coimmunization) (Figure 4E), also confirmed that antigen-driven S1p1 downregulation on specific Co-OVA asTregs impaired their chemotactic capability under a S1p gradient (Figure 4F). These results suggest that asTregs, upon second stimulation by the same antigen in hLN, downregulated their surface S1p1 and no longer heeded the call of S1P in the periphery; and asTregs activated otherwise without this second antigenic hit were not restrained in hLN. To verify that S1p1 reduction on the asTregs was required for the asthma amelioration, we overexpressed S1p1 in asTregs (designated as S1p1^ov^ asTreg) (Figure 4G; Figures S3A,B in Supplementary Material: QC of S1p1 overexpression). After adoptive transfer, the S1p1^ov^ asTregs were no longer retained in hLN (Figure 4H) and failed to suppress asthma (Figure 4I). Therefore, the antigen-triggered S1p1 reduction on the asTregs is crucial for its accumulations in hLN and asthma treatment.

### Antigen-Specific Tregs Control Naïve T Cell Priming and Trap Inflammatory Teff in hLN

**Figure 5:**
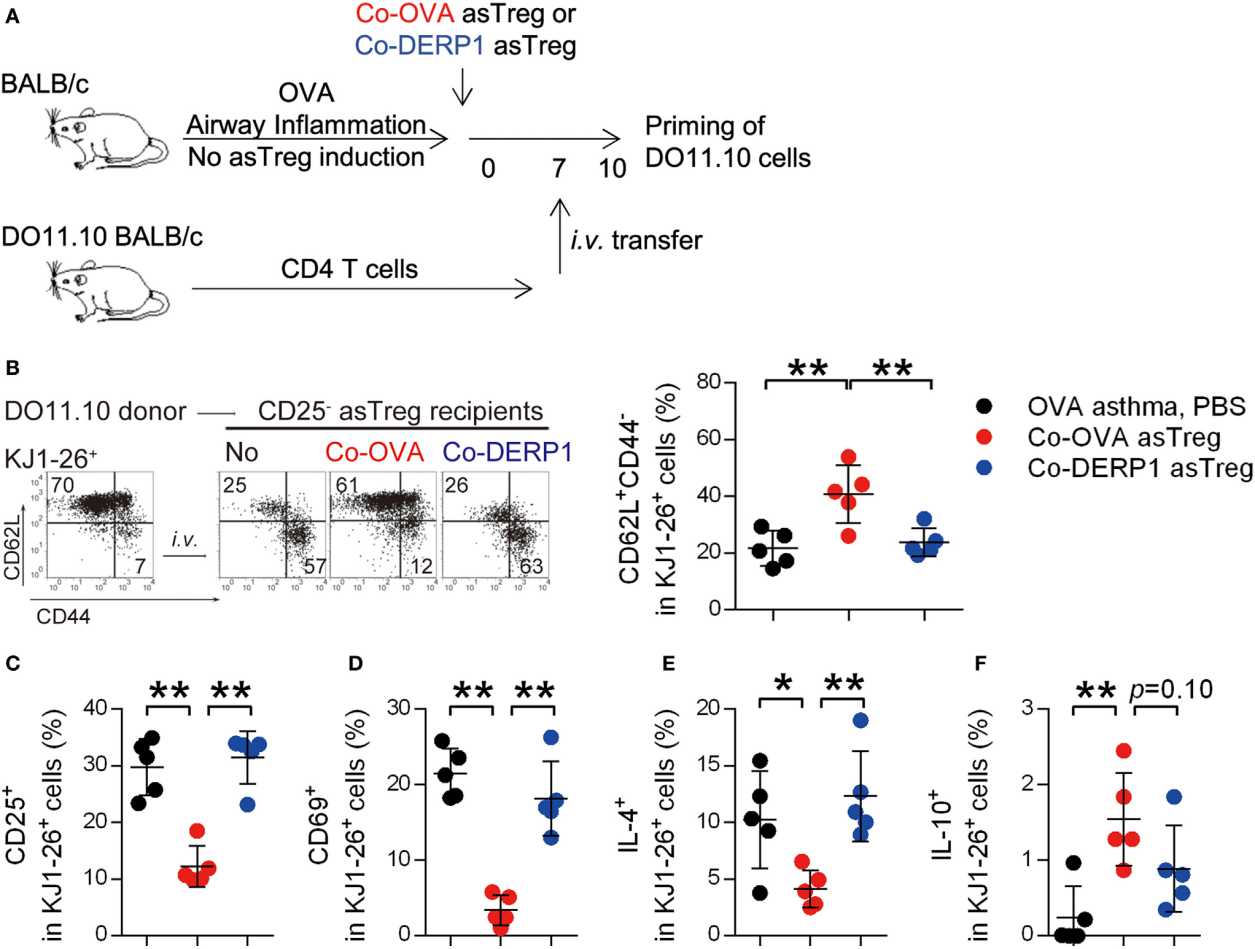
Antigen-specific regulatory T cells (asTregs) control naïve T cell priming in hilar lymph node (hLN). **(A)** Experimental scheme for analyzing naïve T cell priming. DO11.10 spleen CD4^+^ T cells were used to test T cell priming. Asthmatic recipients were transferred asTregs for 7 days, then i.v. infused with donor DO11.10 CD4^+^ T cells. Maturation and polarization of these cells were analyzed within hLN using KJ1-26 as marker. Both antigen-matched (Co-OVA, red) and mismatched (Co-DERP1, blue) were analyzed. **(B)** Phenotype of DO11.10 donor cells in hLN. Statistical analysis is shown in the right panel. *n* = 5. **(C,D)** Activation markers CD25 **(C)** and CD69 **(D)** on DO11.10 cells in hLN. *n* = 5. **(E)** IL-4 expression in DO11.10 cells in hLN. *n* = 5. **(F)** IL-10 expression in DO11.10 cells in hLN. *n* = 5. Data shown represent three independent experiments.

Asthmatic remodeling of the airways is a chronic process: persistent priming of T cells is a crucial underlying cause (53, 54). We analyzed whether Co-OVA asTregs limited airway inflammation by reducing T cell priming in hLN. OVA-sensitized, asTreg-recipient asthmatic mice were infused with cognate DO11.10 CD4^+^ T cells on day 7 after the asTreg transfer, and the maturation and polarization of these T cells in hLN were analyzed 3 days later (Figure 5A). Most KJ1-26 (DO11.10 TCR-clonal) donor cells retained their CD62L^+^CD44^−^ naïve state in Co-OVA asTreg-treated recipients (Figure 5B) and were CD25 and CD69 negative (Figures 5C,D), confirming their resting state. It should be noted that any activated T cells or Tregs might contribute to the suppression by competition for antigen and IL-2 *in vivo*, as was seen in the *in vitro* assay, however the high percentage of resting T cells in the Co-OVA group was indicative of an active suppression. In addition, Th2 polarization of donor cells in hLN was also controlled by specific asTreg treatment as a two- to threefold reduction in the number of IL-4^+^ KJ1-26 cells was seen (Figure 5E). A small (~1.5%) yet statistically significant increase of IL-10^+^ donor T cells was evident in the same treatment group (Figure 5F), collectively indicating a switch to a less pro-inflammatory state in these T cells. Both PBS and non-specific Co-DERP1 asTreg recipients showed activation and polarization of donor DO11.10 T cells (Figures 5B–F), suggesting the priming controlled by Co-OVA asTregs was antigen dependent. These results support the notion that antigen-specific naïve T cell priming is controlled in hLN in the presence of antigen-specific asTregs.

**Figure 6:**
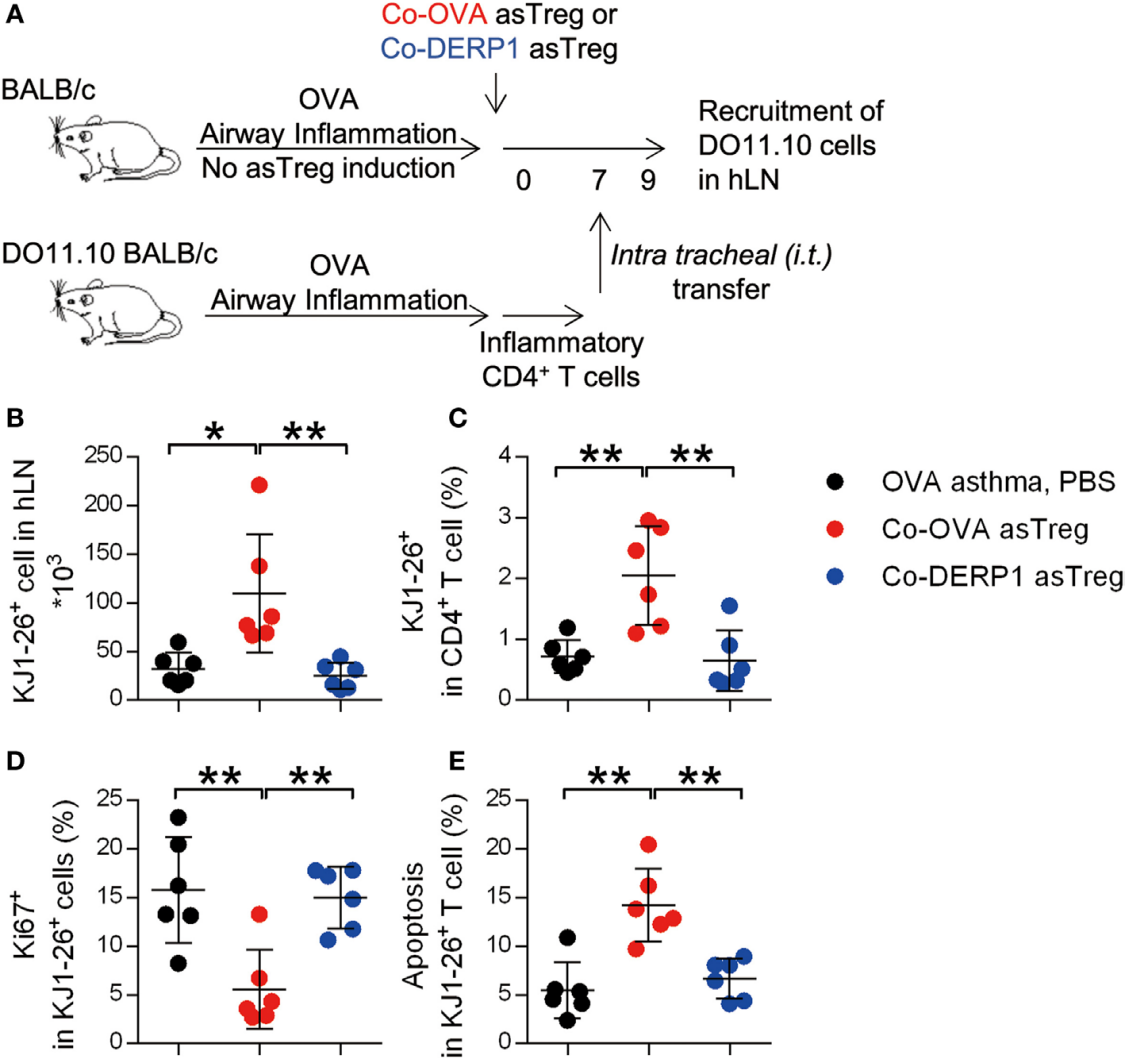
Antigen-specific regulatory T cells (asTregs) trap inflammatory effector T cell (Teff) in hilar lymph node (hLN). **(A)** Experimental scheme to mimic inflammatory infiltration in the lung. DO11.10 mice were sensitized with OVA to build up Th2 inflammation, then spleen CD4^+^ T cells were purified and i.t. transferred into the lung of asTreg-treated asthmatic recipient mice on day 7 (Figure S4 in Supplementary Material). Thirty-six hours later, recruitment of inflammatory DO11.10 cells back to hLN was analyzed. 3 days later, proliferation, apoptosis and cytokine secretion for these inflammatory DO11.10 cells were analyzed in hLN. **(B,C)** Recruitment of DO11.10 cells from the lung to hLN 36 h after i.t. transfer. Absolute number **(B)** and percentage in hLN CD4^+^ cells **(C)** were analyzed with KJ1-26 marker. *n* = 6. **(D,E)** Proliferation (Ki67^+^) **(D)** and apoptosis (AnnexinV^+^PI^−^) **(E)** of DO11.10 cells in hLN 3 days after i.t. transfer. *n* = 6. Data shown represent three independent experiments.

As hLN is the main location of asTreg-mediated suppression, we were curious if effector T cells were also attracted to this location for optimal inhibition, which would also limit the number of those T cells in the lung. DO11.10 CD4^+^ T cells from sensitized mice as Teff were i.t. (intratracheal) transferred into the asTreg-treated OVA asthma model 7 days after the asTreg transfer (Figure 6A; Figure S4A in Supplementary Material). As the baseline without asTreg treatment, within 24 h, half of donor inflammatory T cells infiltrated lung tissues which cannot be washed out as bronchoalveolar lavage (Figure S4B in Supplementary Material). Recruitment of lung-infiltrating DO11.10 Teffs back to recipients’ hLN was detected at 36 h. The number and percentage of DO11.10 Teffs in hLN were increased in allergen-specific Co-OVA asTreg-transferred group (Figures 6B,C). Non-specific asTreg-treated recipient mice showed a lower T cell recruitment to hLN similar to the sham-treated. Importantly this accumulation of Teff also relied on the antigenic match between the suppressor asTregs and the suppressed Teffs.

We analyzed the donor DO11.10 cells’ apoptosis and proliferation, as these factors affected the output of these cells from hLN. As shown in Figure 6, Teffs were less proliferative as measured by Ki67 staining (Figure 6D) and had higher levels of apoptosis (Figure 6E) in the presence of Co-OVA asTregs.

### The Antigen-Driven LN Cohabitation of asTregs and Teffs Is S1p1-Dependent

**Figure 7:**
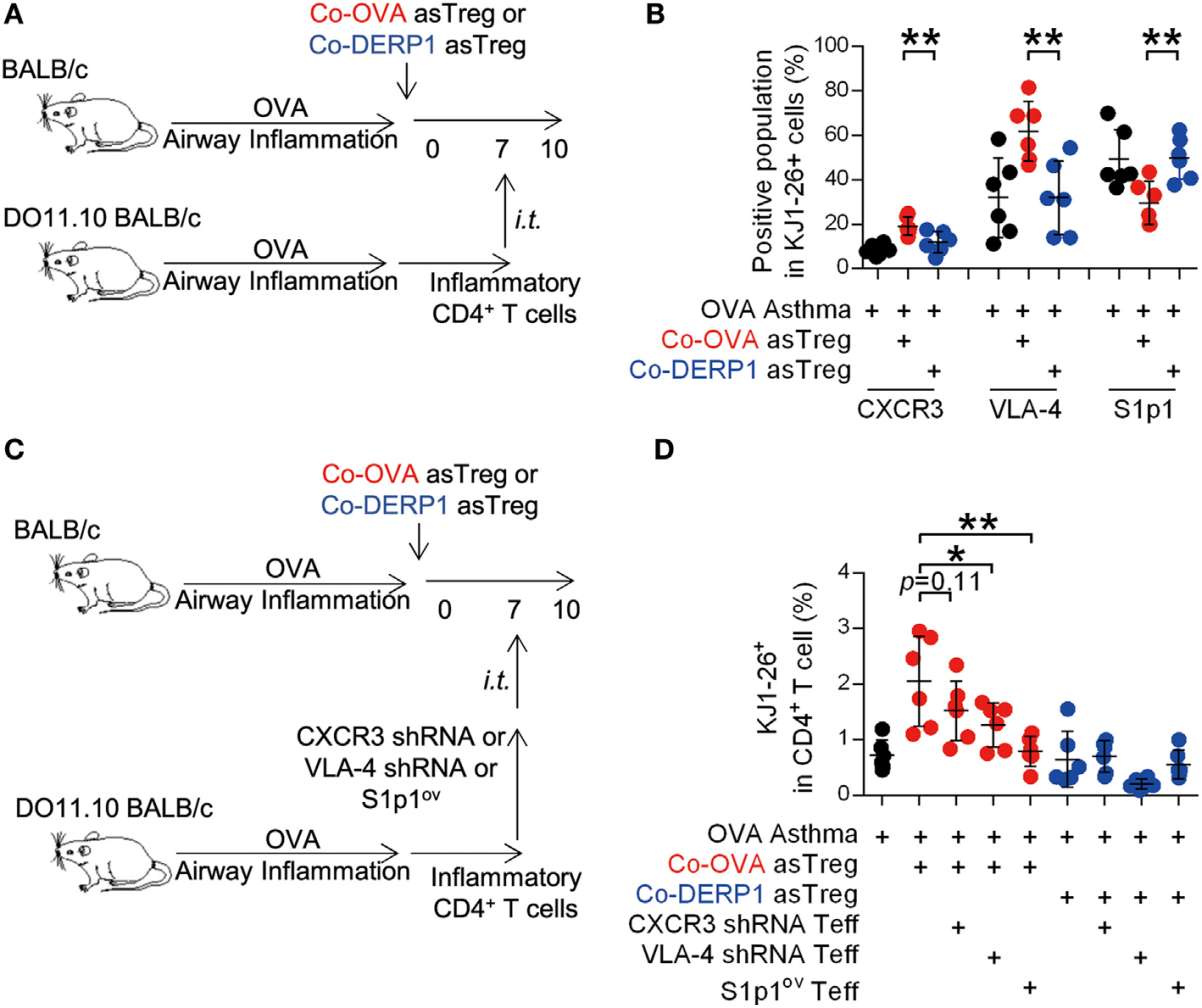
Antigen-specific regulatory T cells (asTregs) control the traffic of inflammatory effector T cells (Teffs) in hilar lymph node (hLN). **(A)** Similar to Figure 6A. 3 days after the i.t. transfer (day 10), chemokine receptors and adhesion molecules were analyzed on KJ1-26^+^ cells in hLN. Both antigen-matched (Co-OVA, red) and mismatched (Co-DERP1, blue) treatments were analyzed. **(B)** Enrichment of the CXCR3^+^, VLA-4^+^, S1p1^−^ population in DO11.10 T cells in hLN. *n* = 6. **(C)** Experimental scheme for lentivirus knockdown assay. CXCR3 and VLA-4 knockdown, and S1p1-overexpression were performed on donor inflammatory DO11.10 T cells, and they were i.t. transferred into asthmatic asTreg-recipient mice on day 7. **(D)** On day 10, KJ1-26^+^ cells recruited back to hLN were analyzed. *n* = 6. Data shown represent two independent experiments.

Mirroring the asTregs, expression profile of ingress chemokine receptors, adhesion molecules and egress chemokine receptor S1p1 on DO11.10 Teffs recovered from the hLN on day 10 was analyzed (Figure 7A). No statistical difference was observed except for significantly increased CXCR3 and VLA-4, as well as a decreased S1p1 (Figure S5 in Supplementary Material and Figure 7B). To verify the role of these molecules in Teff trafficking through hLN in the presence of asTregs, donor DO11.10 Teffs were premodified with CXCR3 and VLA-4-specific short harprin RNA (shRNA) or the vector with S1p1-overexpressing sequence (Figures S3C–E in Supplementary Material). Modified donor cells were transferred and analyzed in parallel experiments (Figures 7C,D). While neither sham nor Co-DERP1 immunized retained Teffs, forced expression of S1p1 enabled the egress of Teffs from hLN (Figure 7D). CXCR3 and VLA-4 knockdowns showed a marginal decrease of Teff retention (Figure 7D). Therefore, while Teff egress, similar to Treg, is mostly regulated by S1p1, they may be additionally controlled by enhanced retention *via* CXCR3 and VLA-4. These observations suggest that these receptors are coordinated for the eventual entrapment of Teffs in hLN.

Together, we identified an antigen-specific S1p1 downregulation on Tregs and effector T cells, preventing their egress from hilar LNs in an allergen-induced asthma model, thereby enabling long-lasting and efficient suppression of effector T cells at this site. This regulatory event in the LN is critical to the amelioration of airway inflammation in the lung.

## Discussion

One of the effector locations of Tregs is LNs (30, 55–59). There has been no direct confirmation whether this anatomic confinement is obligatory for overall systematic immune inhibition nor is the molecular nature of the gathering understood. We report here that in asthma, Treg-mediated suppression strictly requires the coretention of induced antigen-specific asTregs and antigen-specific CD4^+^ T cells. Our findings reveal that Treg/Teff congregation in LNs as an essential requirement for effective suppression.

Lymph nodes as the main location of Treg-mediated suppression, at least for asTregs that are converted at the site of inflammation/active immune response, is supported by indirect evidence. IL-2 is required for Treg maintenance (60, 61) and suppressive capacity (62). In resting state, sporadic Stat5 activation in Tregs is found to be in isolated clusters evenly distributed in LNs. The clusters are also composed of DCs and CD4^+^ T cells that sparingly produce IL-2 in accumulative response to self-antigens (44). As it is hard to imagine an alternative steady source of IL-2, it is logically consistent that a robust suppression takes place in this IL-2-rich environment, although this argument does not suggest an autonomous signaling cascade that forces both Tregs and conventional T cells into LNs.

The ingress of lymphocytes, including Tregs, into fully developed draining LNs requires CCR7 (30). In addition, Treg-related CCR4, CCR8, and CCR9 (63–66), Th2-related CCR3 (67), Th1-related CCR5 and CXCR3 (68, 69), and Th17-related CCR6 (70) are important to each of their respective subsets. Therefore, it is a little surprising that they were not found to be involved in the asTreg accumulation in hLN. This may mainly reflect that their expression is not regulated by antigenic stimulation at this stage. For instance, it has been reported that tTregs first leave the circulation to enter inflamed tissues *via* CCR2, CCR4, CCR5, and P- and E-selectin. Once they are activated, they move to DLNs *via* CCR2, CCR5, and CCR7 (30). These are considered to be factors positively regulating their eventual accumulation in LNs. We do not know how much antigenic-specific stimulation regulates this ingress, although our limited comparison between antigen-matched and mismatched asTreg stimulation failed to reveal a significant TCR-dependent regulation. Instead a double hit of cognate antigen *via* TCR on asTregs was found to mainly regulate S1p1, stopping asTregs at the time of exit. TCR stimulation and Akt activation suppress transcriptional factor Foxo1 phosphorylation (71), and as a result, downstream KLF2 transcription is suppressed and S1p1 is downregulated (52). This pivotal pathway is functional for αβ (72, 73) and γδ T cells (74). One lingering unknown in this study is the collective signaling that retains Teffs in the same draining LNs. The same signaling events (double hit) sufficient to retain asTregs were also available to Teffs, yet they additionally require asTregs for S1p1 downregulation. We are at this stage unable to provide molecular details to explain this subtle discrepancy, although we postulate that signaling in Teffs *via* TCR may be strong enough to support the egress, yet to be restrained by asTregs to allow S1p1 downregulation.

Physiologically, S1p function is multifaceted; it behaves in general as a tone-setter between vascular permeability and lymphocyte egress (75). S1P (lysophospholipid sphingosine 1-phosphate) is synthesized *via* phosphorylation of intracellular sphingosine. Extracellular S1P has at least five G protein-coupled receptors (designated S1p1-5) (76). Among them S1p1 signaling *via* PI3K and Rac GTPases is a robust egress mediator for CD8^+^ and CD4^+^ T cells, controlling their exit from the thymus and secondary lymphoid organs (77). How this rheostat regulation is coupled to immune cell activation is beginning to be understood. In Th2 responses, CD4^+^ cells express extracellular matrix protein-1 (ECM1). ECM1 deficiency in these cells reduces S1p1 expression and leads to their inability to leave LNs (78). Our data reveal that the presence of specific antigen alone is sufficient to trigger asTreg S1p1 downregulation in LNs. Interestingly, it has been reported that S1p1 signaling through Akt-mTOR pathway is a negative regulator of Treg suppressive capacity (79). Therefore, the downregulation of S1p1 as a consequence of TCR stimulation in asTregs will in theory potentiate their suppression.

This report deals with a relatively isolated aspect of Treg regulation: egress control of induced Treg in LNs and its implications in an inflammatory response. Whether this mechanism applies to other types of Tregs or autoimmunity in general needs more comprehensive investigation. In addition, it remains a question whether this regulation is applicable for effector memory T cell migration to draining LNs upon a secondary challenge. In spite of the limitations, revealing chemotaxis/traffic control used by antigen-specific Tregs to suppress inflammation *in vivo*, our report has two implications. In recent years, the focus of Treg suppression has been gradually shifted to a contact-dependent scenario, our results provide a backdrop of how these specific regulations are carried out in anatomically optimal locations. In addition, this regulation may hold therapeutic potential beyond vaccine-based control of asthma. Incidentally, strong clinical and pre-clinical efforts are being made to identify S1P receptor agonists and antagonists (80). FTY720 (Finglimod) (81), an antagonist of S1p1 receptor, has been prescribed to treat multiple sclerosis. In light of the role of S1p1 discussed in this report, Treg functions modulated by these compounds become interesting experimental topics, to explore new disease-intervention methods based on traffic control of Tregs.

## Materials and Methods

### Mice

Foxp3-eGFP/C mice were purchased from JAX. DO11.10 mice were kindly provided by Dr. Minghui Zhang (Tsinghua University, Beijing, China). BALB/c mice were purchased from Vitalriver (Beijing, China). All mice were bred and maintained under SPF conditions at the central animal facility of Shanghai Medical College (SHMC). All protocols of animal experiments are approved by the Committee of Experiment Animals of SHMC.

### Reagents

DNA vaccines (pVAX1-OVA and pVAX1-Der-p1) were produced previously (82) and prepared by EndoFree Plasmid Maxi Kit (Qiagen). His-tagged recombinant Der p 1 protein was prepared by Ni-NTA agarose (Qiagen) and Detoxi-Gel Endotoxin Removing Gel (Thermo). Endotoxin of each vaccine was below 10 EU/mg. All reagents were from Sigma-Aldrich unless specified otherwise. Magnetic CD4^+^ T cell purification kit was purchased from R&D System. LTβR-Ig protein was a kind gift from Dr. Yangxin Fu (University of Chicago) and Dr. Mingzhao Zhu (Institute of Biophysics, Chinese Academy of Science). Lentiviral systems of S1p1 overexpression, CXCR3, and VLA-4 knockdown were gifts from Dr. Ying Wan (Third Military Medical University, Chongqing, China). Antibodies for CCR5 (7A4), CCR7 (4B12), CD44 (IM7), CD62L (MEL-14), CD4 (GK1.5), CD25 (PC61.5), CD69 (H1.IF3), DO11.10 TCR (KJ1-26), IL-35/IL-12p35 (4D10p35), B220 (RA3-6B2), and MHC-II (M5/114.15.2), as well as all ELISA kits, were purchased from eBioscience. Antibodies for CCR3 (TG14), CXCR3 (CXCR3-173), CCR6 (29-2L17), CCR9 (9B6), CD11a (M17/4), and CD49d (R1-2) were from Biolegend. Antibodies for S1p1 (H-60), CCR8 (c-17), and Ki67 (M-19) were from Santa Cruz. Antibody for IL35/EBI3 was from R&D System.

### Flow Cytometry

Single cell suspension was collected from various tissues (hLNs, lungs, and spleens) from sacrificed mice. Surface CD markers were stained at 4°C for 20 min, protected from light. After according treatments, intracellular staining was carried out following surface staining, 4% PFA/PBS fixation and 0.2% TritonX-100 permeabilization. Data were acquired with BD Aria flow cytometer (BD Biosciences, USA) and analyzed by Flowjo (Treestar). Cells were sorted by BD Aria III flow cytometer (BD Biosciences, USA).

### Asthma Model and Treatment

Asthma model as previously reported were modified (33). In brief, mice were i.p. sensitized with 100 µg OVA/alum on day −28 and day −14, followed with OVA spray (1 mg/mL in PBS) at days −7, −4, −1 for 30 min/day/mouse. To treat asthma with coimmunization, OVA + pVAX1-OVA (Co-OVA) or Der p 1 + pVAX1-Der-p1 (Co-DERP1) was i.m. immunized at 100 µg + 100 μg dosage (single dose, in right thigh), on day 0 and day 14. To deplete Tregs *in vivo*, low-dose cyclophosphamide (Cy) was i.v. injected at 20 mg/kg, on day 7 and day 14. To treat asthma with CD25^−^asTregs, Foxp3-eGFP mice (donors) were coimmunized on day −21 and −7. On day 0, GFP^+^ cells were sorted from CD4^+^CD25^−^ population of donor splenocytes, then i.v. transferred into asthmatic mice (recipients) at 3 × 10^5^ asTregs/mouse. On day 14, asthma of recipients was evaluated. Plethysmography was analyzed as reported (33). Briefly, mice were anesthetized and ventilated. Respiratory resistance was stimulated with methylcholine chloride and recorded by AniRes 2005 system (BestLab Technology Co., Beijing, China). Respiratory resistance was analyzed as relative area of peak (R-area) above the baseline before stimulation. To test infiltrating CD4^+^ T cells, lungs were perfused with 10 mL PBS, minced into single-cell suspension and analyzed on flow cytometer. H&E histology was analyzed by counting infiltrated cells in slide image based on nuclei with ImageJ.

### Cell Culture and *In Vitro* Suppression

All culture experiments were performed in DMEM with 10% FBS, at 37°C 5% CO_2_. Sorted asTregs or hLN cells were restimulated with OVA or Der p 1 (10 µg/mL) in the presence of DCs (DC:T = 1:5, 105 T cells/well) for 24 h. Four hours before collection, monensin (BD GolgiStop) and brefeldin A (BD GolgiPlug) were added into the culture to block cytokine secretion. Cells were fixed and permeabilized for intracellular cytokine stain. To evaluate asTreg proliferation, sorted asTregs were labeled with 10 µM eFluor670 (eBioscience) and restimulated with OVA or Der p 1 in the presence of DCs (DC:T = 1:1, 5 × 10^4^ each cells/well) for 3 days, then analyzed as eFluor dilution by flow cytometry. To determine asTregs’ suppression, DO11.10 CD4^+^ T cells were labeled with 10 µM eFluor670, stimulated by OVA (10 µg/mL) with DCs (T:DC = 5:1), then sorted asTregs were added (asTreg:T = 1:5, 5 × 10^4^ asTregs/well). Division was analyzed 3 days later.

### Adoptive Transfer

To test naïve T cell priming, CD4^+^ T cells from naive DO11.10 mice were purified and i.v. transferred into asTreg recipient mice on day 7 at 5 × 10^6^/mouse. On day 10, hLNs were collected and KJ1-26 cells were stained for maturation and polarization. For i.t. transfer of Teffs, CD4^+^ T cell of asthmatic DO11.10 mice was purified with magnetic beads (R&D System). asTreg-transferred asthmatic mice were anesthetized i.p. with 80 mg/kg pentobarbital, and a sterilized cut (~5 mm) was made right above trachea. 10^7^ purified cells were loaded in 50 µl microinjector then intratracheally injected on day 7. After 36 h, hLN cells of recipients were stained and KJ1-26^+^ cells were analyzed.

### Lentivirus

To overexpress S1p1 (NM_007901.3, 1149bp), HIV-based lentiviral expression system was used (Cell Biolabs). S1p1 gene was synthesized from cDNA and cloned into expression vector pSMPUW-IRES-Bsd. Together with packaging plasmids, pSMPUW-IRES-Bsd-S1p1 was transfected into 293FT cells. Lentiviruses were harvested from the culture supernatant, concentrated and added into asTreg suspensions at virus:cell = 3:1 ratio. After 24 h, S1p1 expression on cells were validated by S1p-dependent chemotaxis. Four hours after transfection with validated batch of virus, S1p1 overexpressed (S1p1^ov^) asTregs were i.v. transferred. S1p1 overexpression on Teff followed the same procedure. To knockdown CXCR3 or VLA-4, FIV-based lentivirus coding shRNA was used (System Biosciences). shRNA carried by the pFIV U6/H1 puro vector were cotransfected into 293FT cells with packing plasmids. Lentiviruses were collected and transfected into DO11.10 Teffs at virus:cell = 3:1 ratio. Expression of CXCR3 and VLA-4 was determined by surface stain. IP-10-dependent chemotaxis was checked by transwell migration with CXCR3 shRNA transfected T cells. The shRNA and control sequences were CXCR3 shRNA sense 5′-AAAGTGTGGATGTTGTTCACGCG-3′, antisense 5′-AAAACGCGTGAACAACATCCACA-3′, VLA-4 shRNA sense 5′-AAAGCAATGGATATGTTGATGTA-3′, antisense 5′-AAAATACATCAACATATCCATTG-3′, control shRNA sense 5′-AAAGCTCCGAACGTGTCACGTTT-3′, antisense 5′-AAAAAAACGTGACACGTTCGGAG-3′.

### Peripheral LN Ablation

Lymph node null mice were prepared according to a standard protocol (47). Pregnant female BALB/c mice were i.v. injected with 100 µg of purified murine LTβR-Ig on gestation day 12. To confirm the successful LN ablation, offspring mice were sensitized and challenged to induce asthma, and representative mice were studied for the absence of peripheral LN including hLN, popliteal LN, and axillary LN.

### Microscopy

To determine asTreg distribution in hLN, hLNs of asTreg-transferred recipient asthmatic mice were collected on day 2 after asTreg transfer and frozen in liquid nitrogen immediately. hLNs were mounted in OCT (Tissue-Tek) and 5 µm cryosection were cut with Cryostat (Leica). Slides were fixed with 4% PFA and stained with anti-B220-PE. Images were acquired using the A1R confocal microscopy and NIS-Element AR software (Nikon). Image analysis was done using ImageJ.

### Transwell Migration

10^5^ asTregs (WT or S1p1^ov^) or asthmatic DO11.10 Teff (WT, CXCR3 shRNA or S1p1ov) were added in upper chamber of Transwell 96-well plate (Corning). Medium or 20 nM chemokine S1p (Cayman Chemical) or IP-10 (R&D systems) were added into lower chamber, respectively. Cells were allowed to migrate for 4 h at 37°C. The number of migrated cells was assessed by FACS with 10^5^ calibration beads (Sepherotech) as internal control. Results are expressed as Migration Index = fold increase of specific migration over unspecific migration without chemoattractant.

### Statistics

Data were showed as mean ± SD. Data were analyzed using one-tailed Mann–Whitney *U* test between two groups (Figures 1E,G,H, 2B–E, 3C,D, 4D,F, 5B–F, 6B–E, and 7B,D; Figures S1C,F and S3C in Supplementary Material) and two-way ANOVA for multiple dimensional comparison (Figures 1B,C, 2H,I, 3B, and 4C,G,H). Differences were considered to be statistically significant with *p < 0.05 and **p < 0.01.

## Ethics Statement

All mice were bred and maintained under SPF conditions at the central animal facility of Shanghai Medical College (SHMC). All protocols of animal experiments are approved by the Committee of Experiment Animals of SHMC.

## Author Contributions

SG and BW designed the project. SG, BW, and SY wrote the manuscript. SG, YZ, and XZ built up model system and analyzed the data. XX performed surgery and histology analysis. YP performed respiratory resistance tests. HL, HZ, and GZ constructed and validated vectors.

## Conflict of Interest Statement

The authors declare that the research was conducted in the absence of any commercial or financial relationships that could be construed as a potential conflict of interest.
