# Supplementary material for: Induced Regulatory T Cells Superimpose Their Suppressive Capacity with Effector T Cells in Lymph Nodes via Antigen-Specific S1p1-Dependent Egress Blockage
Source: Front Immunol. 2017 Jun 7;8:663. doi: 10.3389/fimmu.2017.00663 (PMC5461288; doi:10.3389/fimmu.2017.00663)
Supplement: Supplementary file 1 [file image_1.pdf]

Supplementary Material

Induced regulatory T cells superimpose their suppressive capacity with effector T cells in lymph nodes via antigen specific S1p1-dependent egress blockage

Shuang Geng1, Yiwei Zhong1, Xiaoyu Zhou1, Gan Zhao1, Xiaoping Xie2, Yechun Pei2, Hu Liu2, Huiyuan Zhang†, Yan Shi3* & Bin Wang1,*

[*]Corresponding authors

Dr. Bin Wang, bwang3@fudan.edu.cn;

or Dr. Yan Shi, yanshi@biomed.tsinghua.edu.cn.

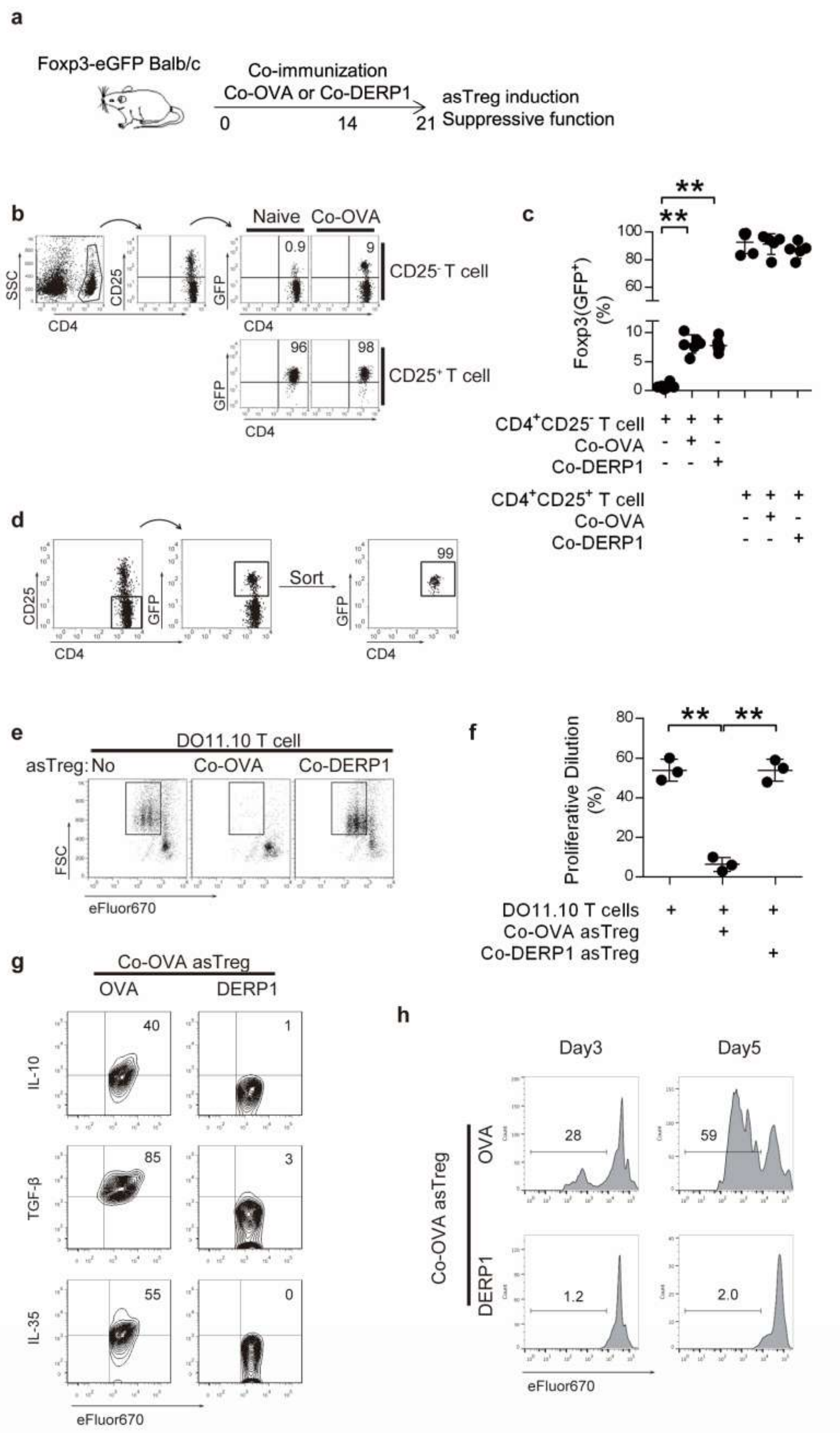
a
Foxp3-eGFP Balb/c
Co-immunization
Co-OVA or Co-DERP1
0
14
21
asTreg induction
Suppressive function
b
SSC
CD4
CD25
Naive
Co-OVA
0.9
9
96
98
GFP
CD25⁻ T cell
CD25⁺ T cell
c
Foxp3(GFP⁺) (%)
CD4⁺CD25⁻ T cell
Co-OVA
Co-DERP1
CD4⁺CD25⁺ T cell
d
Sort
99
e
DO11.10 T cell
asTreg: No
Co-OVA
Co-DERP1
FSC
eFluor670
f
Proliferative Dilution (%)
DO11.10 T cells
Co-OVA asTreg
Co-DERP1 asTreg
g
Co-OVA asTreg
OVA
DERP1
IL-10
TGF-β
IL-35
40
1
85
3
55
0
h
Day3
Day5
28
59
1.2
2.0

Supplementary Figure 1. Co-immunization induce functional CD25$^-$ asTregs

(**a**) Naïve Foxp3-eGFP transgenic mice were co-immunized with Co-OVA on day -21 and -7, phenotypical and function analyses were performed on day 0. (**b**) asTreg induction by co-immunization. Naïve mice splenocytes were stained as control. (**c**) Statistical analysis of asTreg induction. n=5. (**d**) Sorting efficiency of CD25- asTreg is routinely>98%. (**e**) Suppressive function of asTregs *in vitro*. DO11.10 CD4$^+$ splenocytes were purified and labeled with eFluor670, then *in vitro* stimulated with OVA presented by DCs for 3 days. Negative control was set up without asTreg co-culture. (**f**) Statistical analysis of *in vitro* suppression experiment. n=3. (**g**) FACS data of asTreg cytokine secretions after *in vitro* restimulation . (**h**) Proliferation of asTregs after *in vitro* restimulation . Data shown represent 3 independent experiments.

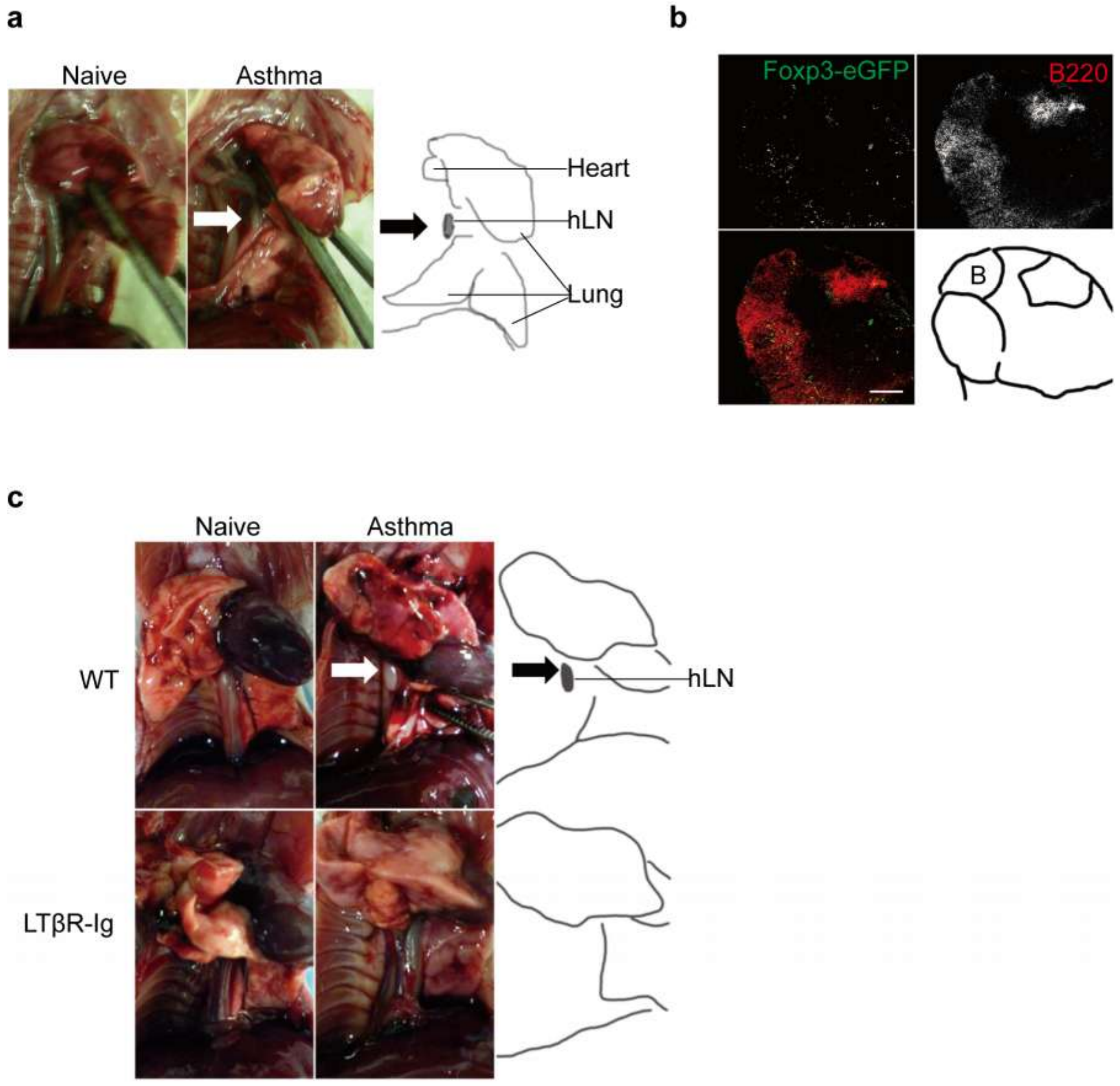

Supplementary Figure 2. Hilar lymph node anatomy and ablation

(**a**) Physical location of lung-draining hilar lymph node (hLN). Naïve mice have no visible hLN, whereas asthmatic mice showed expanded hLN 1~3mm in diameter. (**b**) asTreg distribution within hLN. Cryo-sections of hLN were stained for B220. 200µm scale. (**c**) Maternal LTβR-Ig injection ablates hLN (lower-right).

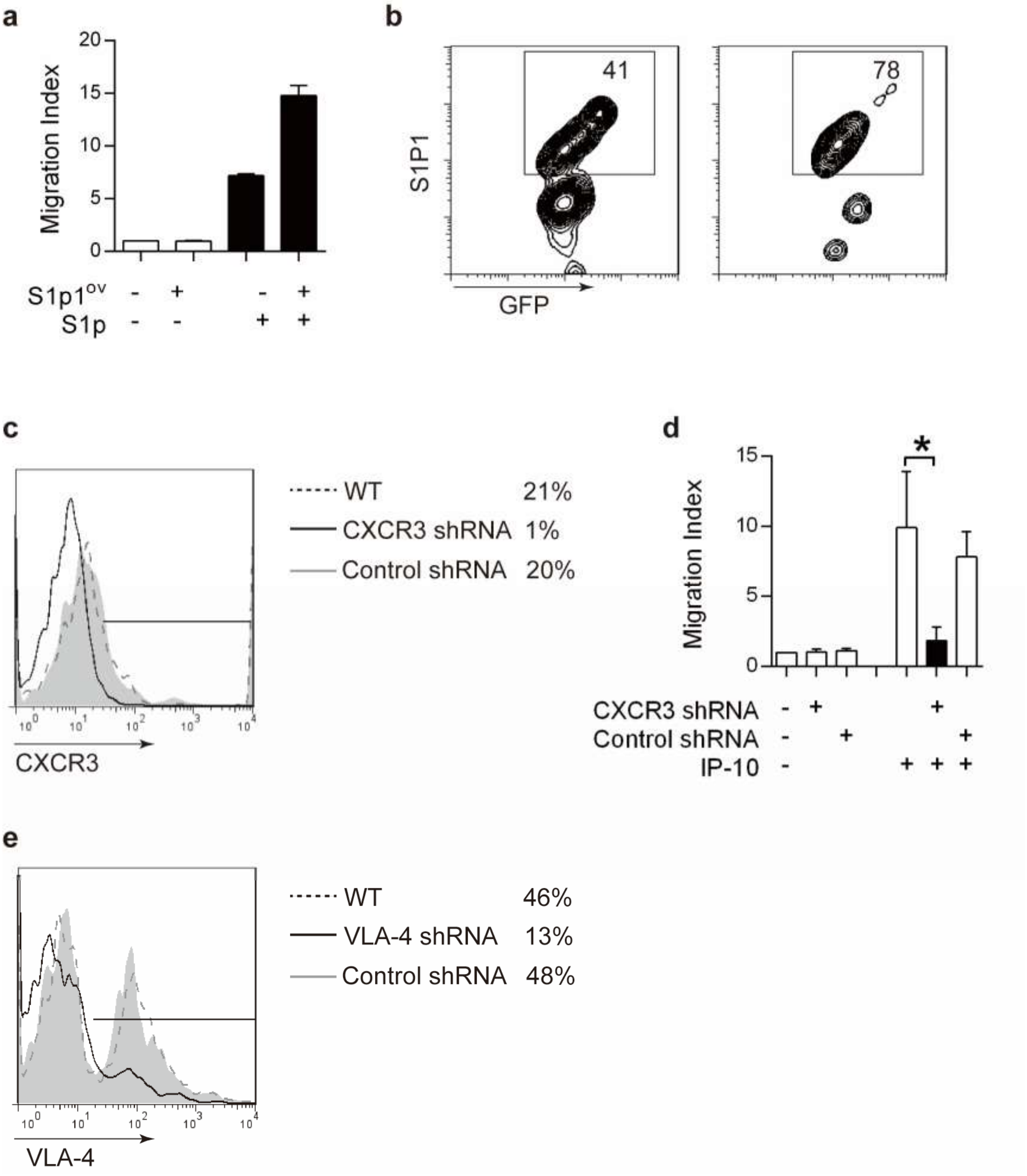

Supplementary Figure 3. S1p1 over-expression, and CXCR3 and VLA-4 knock-down

(**a-b**) Sorted asTregs were transfected with lentivirus coding S1p1 gene. (a) S1p chemotaxis of donor cells. WT or S1p1 over-expressing (S1p1^{ov}). (b) S1P1 expression before (left) and after (right) overexpression. n=3. (**c-e**) Donor inflammatory DO11.10 Teff cells were transfected with lentivirus coding CXCR3, VLA-4 or control shRNA. (**c**) CXCR3 surface stain of the transfected donor cells. (**d**) IP-10 induced transwell of transfected donor cells. n=3. (**e**) VLA-4 surface stain of transfected donor cells. n=3. Data shown represented 3 independent experiments.

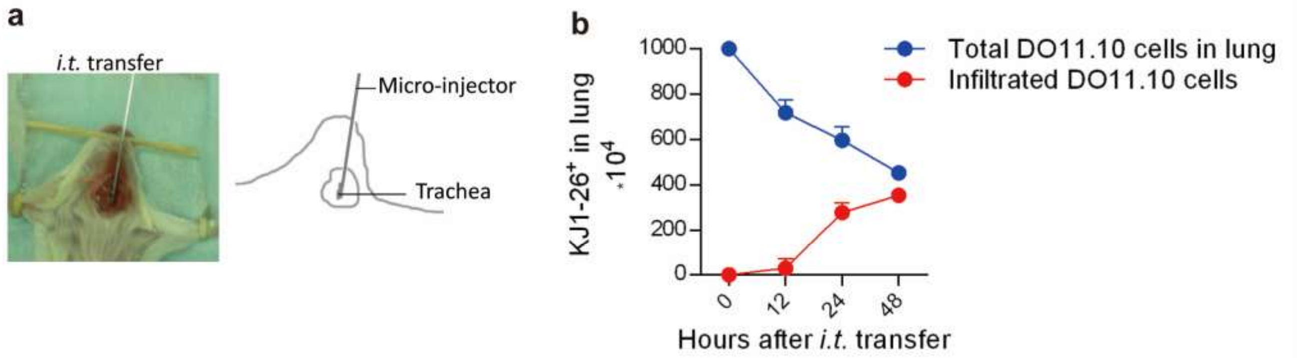

Supplementary Figure 4. Intra-tracheal transfer of inflammatory DO11.10 cells

(**a**) Surgical procedure of intra-tracheal (*i.t.*) transfer. 10^7 donor cells were injected into recipient lung in 50 ul PBS with micro-injector. Detailed information is provided in Materials and Methods. (**b**) Different time points after i.t. transfer, lungs were perfused by cardiac PBS perfusion to remove blood. Non-infiltrated cells were defined as those cells could be washed out in bronchoalveolar lavage (BAL). Infiltrating cells (red) were defined as those cells remained after 3-time BAL washing. Total cells were shown (blue). n=3. Data shown represent 3 independent experiments.

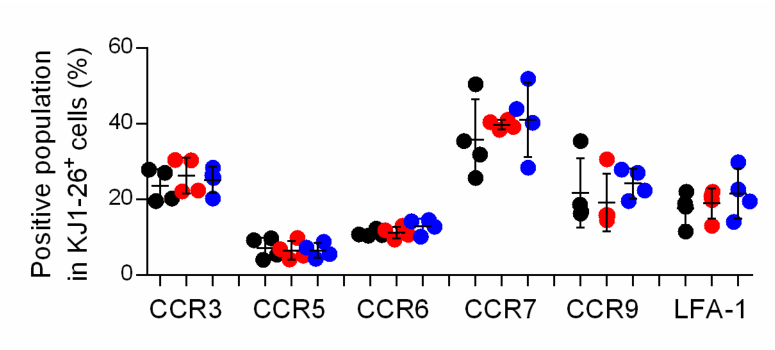

Supplementary Figure 5. Chemokine receptor and adhesion molecule phenotype on DO11.10 inflammatory donor cells in hLN

Experimental scheme for inflammatory infiltration is the same as Fig. 5. Both antigen-matched treatment (Co-OVA, red) and mismatched (Co-DERP1, blue) by asTregs were performed on OVA asthmatic recipient mice. PBS sham-treatment recipient mice were compared as control (black dot). CCR3, CCR5, CCR6, CCR7, CCR9 and LFA-1 expression on donor inflammatory DO11.10 T cells in hLN at day 10. n=4. Data shown represent 2 independent experiments.
